# Hyperthermia Inhibits Growth and Stemness of Esophageal Squamous Cell Carcinoma Cells Through Promoting Degradation of GLI1

**DOI:** 10.1155/sci/7249890

**Published:** 2025-12-11

**Authors:** Hui Qin, Xiaole Li, Shichao Duan, Shenglei Li, Minghua Ren

**Affiliations:** ^1^ Department of Pathology, First Affiliated Hospital of Zhengzhou University, Zhengzhou, China, zzu.edu.cn; ^2^ Department of Radiology, First Affiliated Hospital of Zhengzhou University, Zhengzhou, China, zzu.edu.cn; ^3^ Department of Ophthalmology, Henan Provincial People’s Hospital, Zhengzhou University People’s Hospital, Zhengzhou, China, hnsrmyy.net

**Keywords:** cancer stem cell, esophageal squamous cell carcinoma, GLI1, heat shock, hyperthermia, ubiquitination

## Abstract

**Background:**

Hyperthermia is a widely used adjunct treatment for different cancers. The GLI1 is upregulated in ESCC and its expression is associated with the stemness of ESCC.

**Objective:**

We hypothesized that GLI1 constitutes an important hyperthermia treatment target, and investigated its contribution to hyperthermia responses in ESCC.

**Methods:**

The growth of the human ESCC cell lines KYSE70 and KYSE140 was analyzed using CCK‐8, clonogenicity and spheres formation assays after 43°C hyperthermia, under conditions of knockdown or overexpression of GLI1. Stemness‐related proteins were determined using Western blotting and immunofluorescence staining. Last, the molecular mechanism of GLI1 degradation was studied using chemical inhibitors and immunoprecipitation assays.

**Results:**

Hyperthermia increased the ubiquitination and proteasomal destruction of GLI1, causing a rapid decline in GLI1 protein levels of ESCC cells. Similar to GLI1 knockdown, ESCC cells treated with hyperthermia showed growth inhibition associated with the downregulation of cancer stemness proteins.

**Conclusion:**

Our study reveals that hyperthermia can readily destabilize GLI1 levels in ESCC cells and inhibit ESCC cells growth. This proposes new strategies for implementing hyperthermia to target GLI1 driven cancers to improve therapeutic efficacy.

## 1. Introduction

Esophageal cancer is the eighth most common cancer type worldwide and constitutes the sixth leading cause of cancer deaths [1]. The cause of esophageal cancer is complex, genetic mutations are not the sole contributing factor [2], and the treatment options for this condition are not thoroughly established, necessitating further research and development. Although diagnosing it presents challenges, patients with esophageal squamous cell carcinoma (ESCC) are often diagnosed in advanced stages, resulting in a high mortality rate and unfavorable prognosis. It has been reported that hedgehog pathway regulated esophageal homeostasis [3], and was activated in partial esophageal cancer and high GLI1 level correlates with poor prognosis [4–6]. Target hedgehog‐GLI1 pathway can be a powerful therapy strategy for esophageal cancer [7].

Hedgehog pathway has been found activated in many types of cancers, such as breast cancer, esophageal cancer, lung cancer, liver cancer and correlates with poor prognosis, immunotherapy response, radiotherapy resistance, and chemotherapy resistance [8–10]. GLI1 is the key downstream molecular of hedgehog signaling, which is a transcription factor and regulates cancer cell stemness, proliferation, metastasis, and antiapoptosis [11–13]. Target GLI1 is a powerful cancer therapy strategy and many agents that target GLI1 are on preclinical trials stage [14].

A subpopulation of cancer cells, the cancer stem cells (CSCs), is considered to be of particular significance for cancer initiation, progression, and metastasis. CSCs are considered in particular to be therapy‐resistant and may drive disease recurrence [15, 16]. Hedgehog/GLI1 pathway regulates CSCs, so repression of GLI1 is one way to anticancer therapy [16, 17].

Heat shock is a common physiological and pathological stress that has been well studied [18]. The activation of heat shock factor (HSF) is pivotal in inducing heat shock proteins (HSPs), which serve to safeguard cells against heat stress. Under heat shock conditions, some proteins may misfold, prompting HSPs to bind to these proteins and facilitate their degradation and clearance via the ubiquitination–proteasome pathway, thereby preserving cellular integrity [19–21].

Hyperthermia is a therapeutic procedure in which cancer is treated by high temperature in the tumor tissue. In certain cancers, hyperthermia is applied as an adjunct treatment with various established cancer therapy strategies, such as radiotherapy and chemotherapy [22–24]. The rationale for hyperthermia is based on the cell‐killing effects of high temperature, which can inhibit viability of cancer cell by degrading some bad proteins, inhibiting angiogenesis, inducing metabolic reprogram, and activating immune response [25–27].

In this study, we find hyperthermia can cause GLI1 degradation and repress stemness of ESCC cells in vitro. Heat shock can promote GLI1 binds to HSPs, increase GLI1 ubiquitination level and degrade GLI1 through proteasome pathway.

## 2. Materials and Methods

### 2.1. Bioinformatics Analysis

TCGA data used in this study were download from the website: https://gdc.cancer.gov/about-data/publications/pancanatlas.

### 2.2. Cell Culture

Human ESCC cell line KYSE70 and KYSE140 were obtained from the ATCC and cultured in RPMI 1640 medium (SparkJade) at 37°C in an incubator with 5% CO_2_. All basic mediums were supplemented with 1% antibiotics (SparkJade) and 10% fetal bovine serum (FBS).

### 2.3. Hyperthermia

Heat treatments were conducted in a pre‐equilibrated water bath at 43°C. Cells were first changed to preheated medium before semi‐submerging the cell culture dishes for 30–60 min indicated.

### 2.4. Western Blot

Detail procedures were described in our previous papers. Cells or tissues were lysed in RIPA buffer supplemented with protease inhibitors. When protein samples were extracted and protein concentrations were measured by the bicinchoninic acid (BCA) method (Beyotime, P0010S, China), they were diluted in 2× sodium dodecyl sulfate (SDS) loading buffer supplemented with 0.5% β‐mercaptoethanol and boiled for 10 min. The protein samples were separated on SDS‐PAGE gels and transferred to nitrocellulose membranes. The resulting blots were blocked with blocking solution (Kermey Biotech, Zhengzhou, China) for 10 min and next incubated with the indicated primary antibodies diluted with antibody dilution buffer (Kermey Biotech, Zhengzhou, China) overnight at 4°C. Then the blots were washed (3× for 10 min each time) in TBST. Goat anti‐rabbit IgG (1:10,000, Proteintech, SA00001‐2) and Goat anti‐mouse IgG (1:10,000, Proteintech, SA00001‐1) were used as the secondary antibodies. After washing with TBST (3 times for 10 min each), detection was performed using a chemiluminescent HRP substrate (Yamei Luminescent Solution, China) to visualize antibody binding. Co‐IP experiments were performed according to the instructions. The harvested cells were lysed in low‐salt Co‐IP lysis buffer for 1 h at 4°C for IP and Western blotting. Cell lysates were incubated with magnetic beads (10 μL Myc magnetic beads) (MCE, HY‐K0206) for 3 h at 4°C. After washing with the elution buffer, the protein complexes were boiled and subjected to Western blotting.

### 2.5. Antibodies

The antibodies information were as follows: GLI1 (1:1000, Cell Signaling Technology, 3538), OCT4 (1:1000, Proteintech, 11263‐1‐AP), SOX2 (1:1000, ProteinTech, 1‐1064‐1‐AP), NANOG (1:1000, Proteintech, 14295‐1‐AP), ALDHA1A1 (1:1000, Proteintech, 15910‐1‐AP), HSP70 (1:1000, Proteintech, 10995‐1‐AP),GAPDH (1:1000, Proteintech, 10494‐1‐AP), Ubiquitin (1:1000, Huabio, ET1609‐21), Myc tag (1:1000, Huabio, R1208‐1).

### 2.6. shRNA Sequences

Control shRNA sequences are as follows:

Control shRNA F：CCGGTTCTCCGAACGTGTCACGTCTCGAGACGTGACACGTTCGGAGAATTTTTG;
Control shRNA R：AATTCAAAAATTCTCCGAACGTGTCACGTCTCGAGAACGTGACACGTTCGGAGAA.

GLI1 shRNA sequences are as follows:

shRNA1 F: CCGGTACATCAACTCCGGCCAATAGCTCGAGCTATTGGCCGGAGTTGATGTATTTTTG;
shRNA1 R: AATTCAAAAATACATCAACTCCGGCCAATAGCTCGAGCTATTGGCCGGAGTTGATGTA.
shRNA2 F: CCGGGCTCAGCTTGTGTGTAATTATCTCGAGATAATTACACACAAGCTGAGCTTTTTG;
shRNA2 R: AATTCAAAAAGCTCAGCTTGTGTGTAATTATCTCGAGATAATTACACACAAGCTGAGC.

### 2.7. Cell Proliferation

Cell proliferation was assessed using CCK‐8, colony formation and spheres formation assays. For CCK‐8 assays, 96‐well plates were seeded with 4000 cells/well and after the specified times, 10 μL of CCK‐8 solution (SparkJade, CT0001‐B) was added per well and the plates further incubated at 37°C for 1 h. OD values at 450 nm were measured using a spectrophotometer (ThermoFisher Varioskan LUX) and the values normalized for analysis. For colony formation assays, six‐well plates (coated) were seeded with 1500 cells per well and then incubated until the number of cells in single colonies equaled or exceeded 50 cells. The wells were then washed twice with cold PBS, fixed for 15 min in 4% formaldehyde, and stained 10 min with 0.1% crystal violet. Thereafter, the wells were imaged, and the number of total colonies were counted. For spheres formation assays, six‐well plates (uncoated) were seeded with 2000 cells per well and then incubated until the number of cells in single sphere exceeded 150 cells, then the spheres were imaged and collected to do immunofluorescence staining. When studying the effect of heat treatment on cell proliferation, cells underwent heat treatment every 2 days.

### 2.8. Statistical Analysis

All data were statistically analyzed with GraphPad Prism 8.0. Data were determined by unpaired student’s *t*‐test (2 groups comparison), or one‐way analysis of variance (ANOVA; 3 or more group comparisons). Pearson’s test was applied to determine the correlation between clinicopathological parameters and gene expression levels. Data were presented as mean ± SD. Differences with *p*  < 0.05 were considered statistically significant.

## 3. Results

### 3.1. *GLI1* Is Upregulated in ESCC and Correlated With ESCC Progression

Based on TCGA data described in method, we found GLI1/Hedgehog signaling is hyper‐activated in ESCC (Figure 1A). GLI1 expression positively correlates with SOX2 levels (Figure 1B), a well‐known marker of CSCss. Additionally, GLI1 levels show positive correlations with mesenchymal markers, including *SNAI2* (Figure 1C) and *TWIST1* (Figure 1D). The epithelial–mesenchymal transition (EMT) is known to facilitate cancer metastasis.

Figure 1
*GLI1* is upregulated in ESCC and correlated with ESCC progression. Analyze TCGA database described in method. (A) *GLI1* is significantly upregulated in ESCC. (B, C, D) *GLI1* levels are positively correlated with *SOX2* (B), *SNAI2* (C) and *TWIST1* (D) levels respectively. Data are means ± SDs, unpaired student’s *t*‐test. Pearson’s test was applied to determine the correlation between clinicopathological parameters and gene expression levels.

**Figure:**
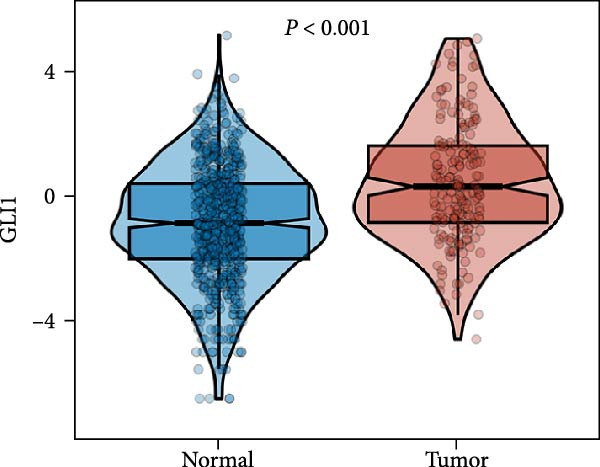
(A)

**Figure:**
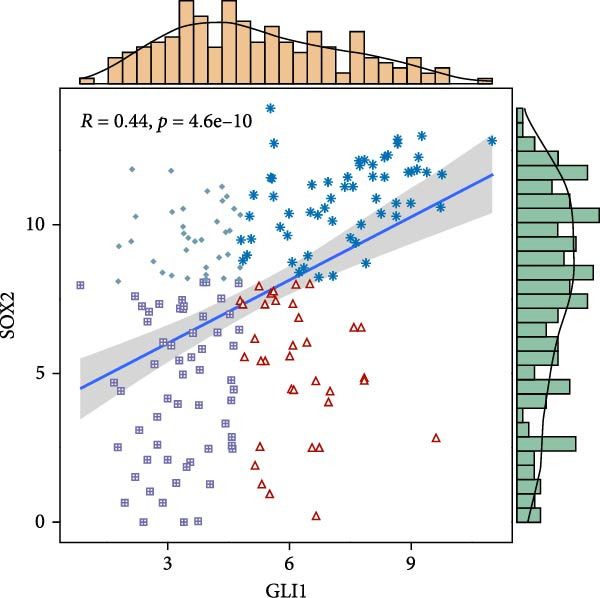
(B)

**Figure:**
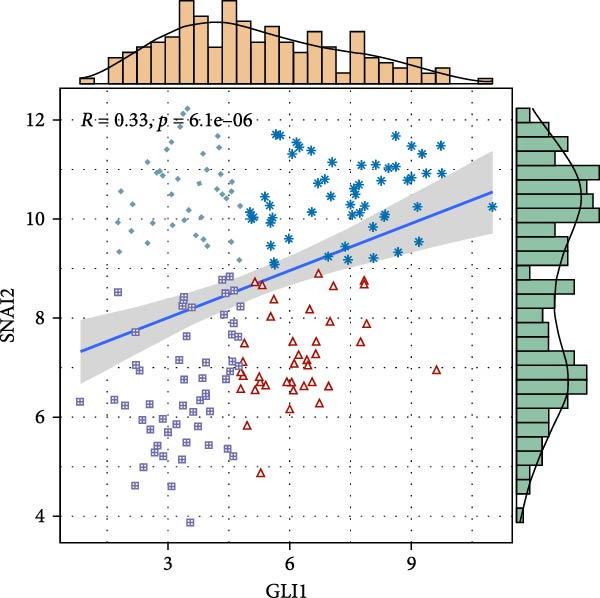
(C)

**Figure:**
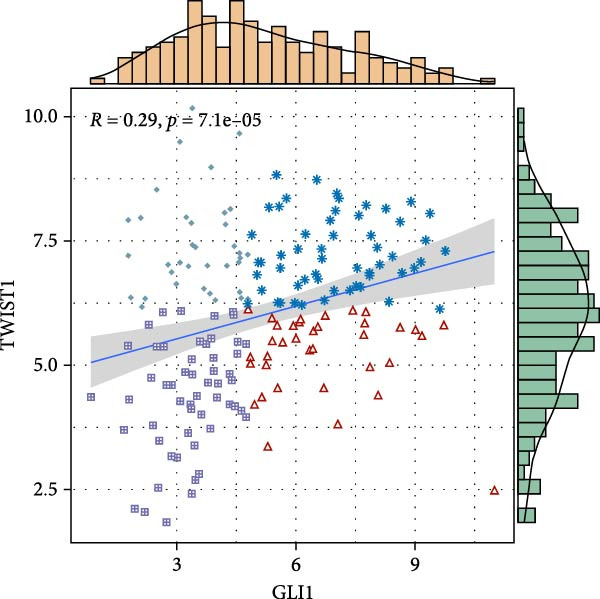
(D)

### 3.2. Knock Down *GLI1* Inhibits ESCC Cell Line Growth and Stemness In Vitro

KYSE70 and KYSE140 expressed higher GLI1 levels than other ESCC cell lines, so we selected KYSE70 and KYSE140 in this study. To prove that GLI1 is essential for ESCC cell growth and stemness, we used shRNA to knockdown *GLI1* expression in the ESCC‐derived cell lines, KYSE70 and KYSE140. Relative to the control shRNA (shCon) group, Western blot assays verified that GLI1 was significantly downregulated, using two independent shRNAs targeting *GLI1* (Figure 2A). Further analysis of cell proliferation ability using CCK‐8 assays demonstrated that *GLI1* knock down significantly inhibited cell proliferation in both KYSE70 and KYSE140 cells after 96 h (*p*  < 0.05) (Figure 2B). Similarly, colony formation assays revealed that *GLI1* knockdown decreased the number of colonies formed by KYSE70 and KYSE140 cells compared to the shCon group (Figure 2C). Since spheres formation assay is a tool to detect stemness of cancer cells, our data revealed that spheres number and size both significantly decreased in *GLI1* knockdown group (Figure 2D,E). Immunofluorescence assays showed SOX2 levels were significantly decreased in spheres of GLI1 knock down group (Figure 2F).

Figure 2Knockdown *GLI1* represses growth and stemness of ESCC cell lines. (A) Western blot analysis of GLI1 levels of indicated cells with *GLI1* knock down or control. (B) CCK‐8 assay found knock‐down *GLI1* can inhibit growth of KYSE70 and KYSE140 cells. (C) Quantification of colony formation assay of indicated cells with *GLI1* knock down or control. (D, E) Representative images of spheres of indicated cells with *GLI1* knock down or control (D). Quantification of spheres number and size of indicated cells (E). (F) Representative immunofluorescence images to detect SOX2 (green) levels in spheres after knock down *GLI1*. Cell nuclei were stained with DAPI (blue), SOX2 and DAPI also been show as gray. Three independent experiments. Data are means ± SDs,  ^∗^ *p* < 0.05;  ^∗∗^ *p* < 0.01;  ^∗∗∗^ *p* < 0.001, one‐way ANOVA.

**Figure:**
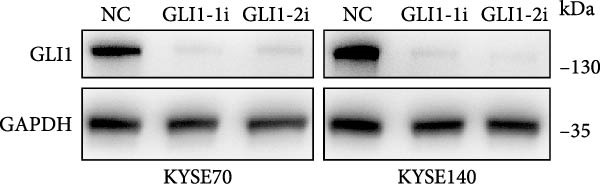
(A)

**Figure:**
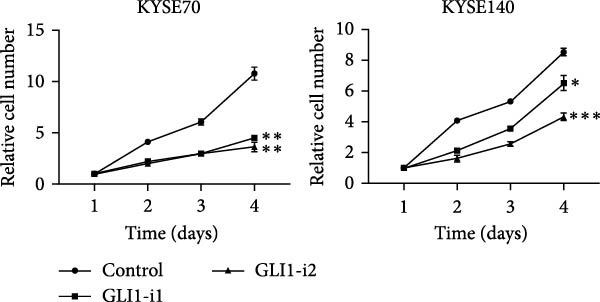
(B)

**Figure:**
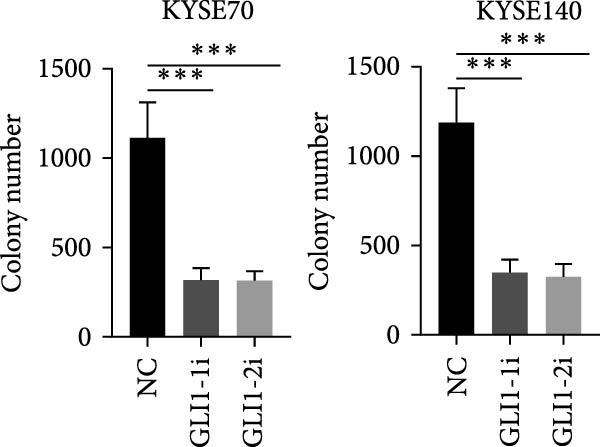
(C)

**Figure:**
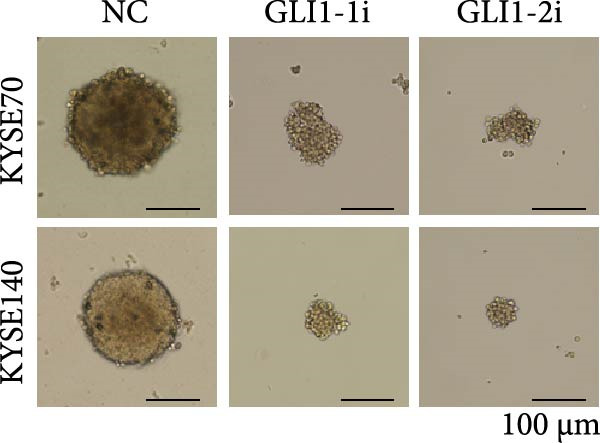
(D)

**Figure:**
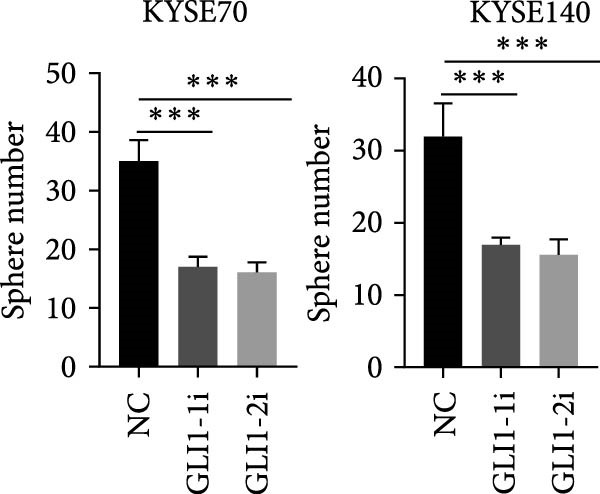
(E)

**Figure:**
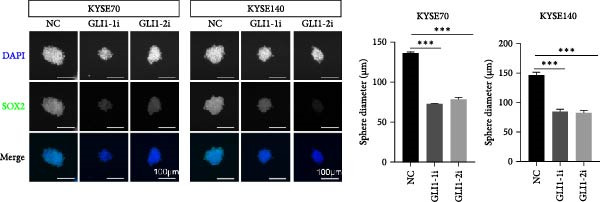
(F)

### 3.3. Overexpressing GLI1 Increases ESCC Cell Line Growth and Stemness In Vitro

To further clarify the effect of GLI1 on ESCC cells growth and stemness, we overexpressed GLI1 in ESCC cell lines using lentiviral mediated transduction. The results of Western blot assay confirmed that GLI1 protein levels were substantially increased in both KYSE70 and KYSE140 cell lines (Figure 3A). Assessing the effects of GLI1 overexpression using CCK‐8 and colony formation assays showed that GLI1 significantly promoted cell proliferation in ESCC cells (Figure 3B,C). Spheres formation assay data revealed that spheres number and size both significantly increased in GLI1 overexpression group (Figure 3D,E). Immunofluorescence assays showed SOX2 levels significantly increased in spheres of GLI1 overexpression group (Figure 3F).

Figure 3Overexpression GLI1 promotes growth and stemness of ESCC cell lines. (A) Western blot analysis of GLI1 levels of indicated cells with GLI1 overexpression or control. (B) CCK‐8 assay found overexpression GLI1 promotes growth of KYSE70 and KYSE140 cells. (C) Quantification of colony formation assay of indicated cells with GLI1 overexpression or control. (D, E) Representative images of spheres of indicated cells with GLI1 overexpression or control (D). Quantification of spheres number and size of indicated cells (E). (F) Representative immunofluorescence images to detect SOX2 (green) levels in spheres after GLI1 overexpression. Cell nuclei were stained with DAPI (blue), SOX2 and DAPI also been show as gray. Three independent experiments. Data are means ± SDs,  ^∗^ *p* < 0.05;  ^∗∗∗^ *p* < 0.001, unpaired student’s *t*‐test.

**Figure:**
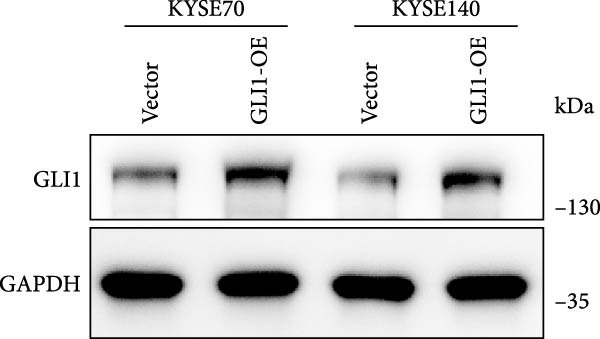
(A)

**Figure:**
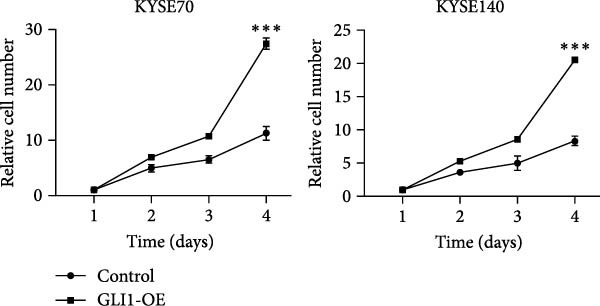
(B)

**Figure:**
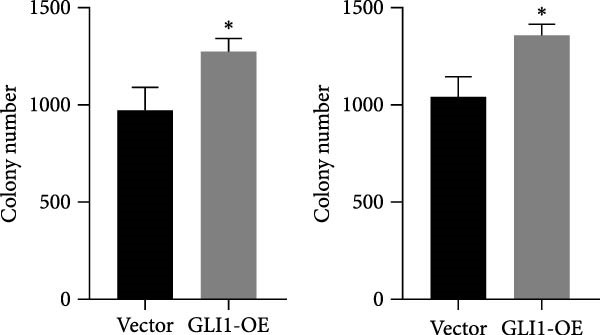
(C)

**Figure:**
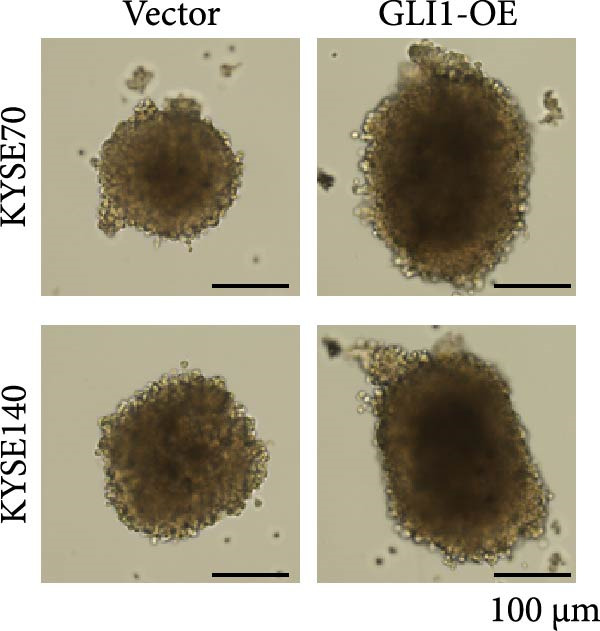
(D)

**Figure:**
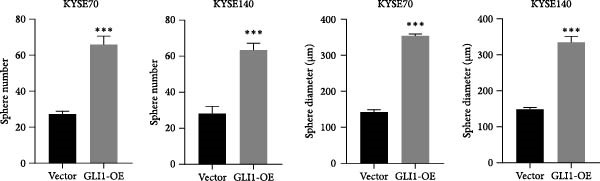
(E)

**Figure:**
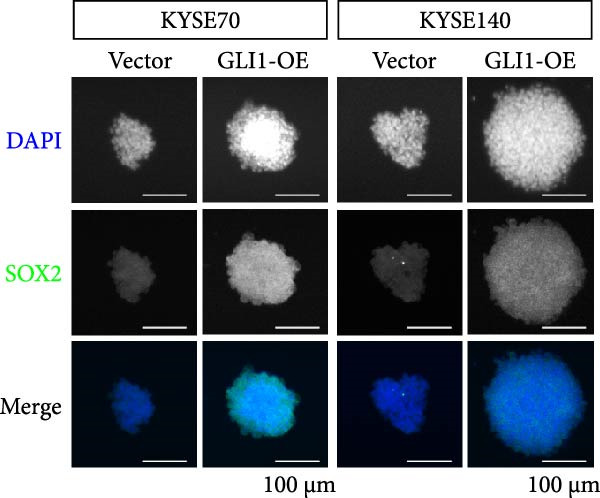
(F)

### 3.4. Hyperthermia Inhibits ESCC Cell Growth Through Targeting GLI1

We wonder whether hyperthermia can inhibit hedgehog signaling or cause GLI1 degradation. In order to mimic hyperthermia in clinic, we treated ESCC cells with 43°C water bathing for different time, harvested samples immediately and analyzed GLI1 protein level using Western blot. Strikingly, we found GLI1 degradation with 15 min treat and the max decrease achieved at 30 min (Figure 4A). CCK‐8 and colony formation assays showed heat shock represses growth of ESCC cells in vitro, cells were treated once (30 min each time) every 2 days (Figure 4B,C).

Figure 4Hyperthermia inhibits ESCC cell lines growth and stemness via causing GLI1 degradation. (A) Effect of 43°C heat treatment on GLI1 protein levels of KYSE70 and KYSE140 cells was measured using Western blot. (B) Effect of 43°C heat treatment on cell proliferation of KYSE70 and KYSE140 cells was measured using CCK‐8 assays. (C) Colony number counts of colony formation assay compared between the Control and heat treatment (HT) groups. (D, E) Representative images of spheres of indicated cells with heat treatment groups or control (D). Quantification of spheres number and size of indicated cells (E). (F) Representative immunofluorescence images to detect SOX2 (green) levels in spheres after heat treatment. Cell nuclei were stained with DAPI (blue), SOX2 and DAPI also been show as gray. (G) Western blotting detected levels of GLI1 and cancer stem cell markers after heat treated in KYSE70 and KYSE140 cells. Cells were heat treated every 2 days. Three independent experiments. Data are means ± SDs,  ^∗∗^ *p* < 0.01;  ^∗∗∗^ *p* < 0.001, unpaired student’s *t*‐test.

**Figure:**
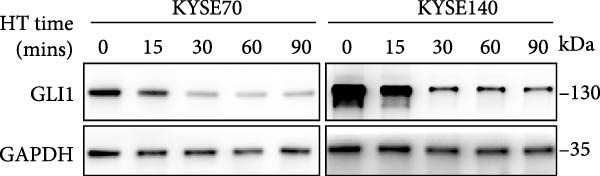
(A)

**Figure:**
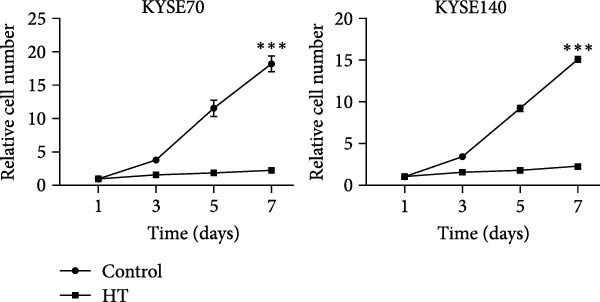
(B)

**Figure:**
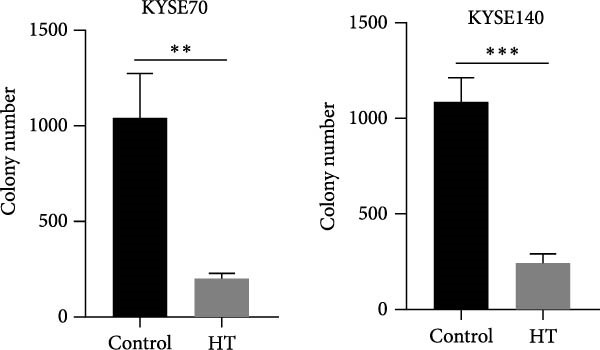
(C)

**Figure:**
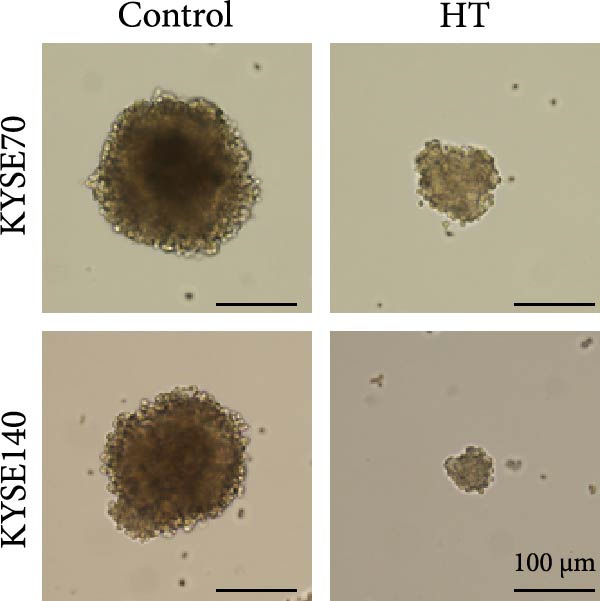
(D)

**Figure:**
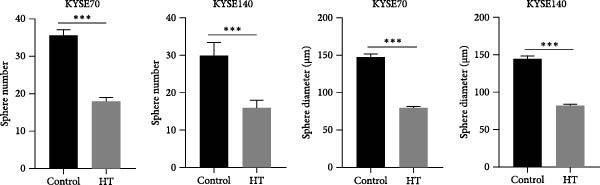
(E)

**Figure:**
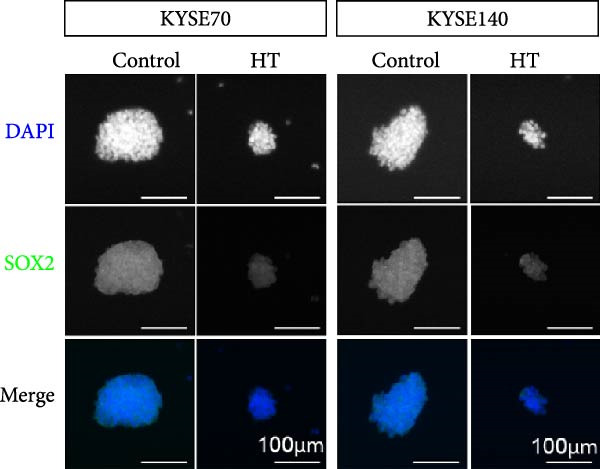
(F)

**Figure:**
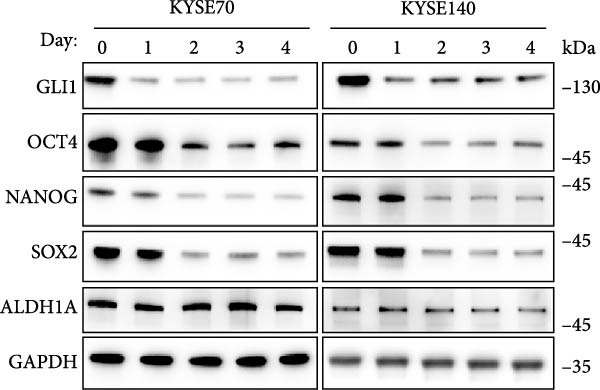
(G)

Spheres formation assay data revealed that spheres number and size both significantly decreased in heat shock group compared with control group (Figure 4D,E). Immunofluorescence assays showed SOX2 levels significantly decreased in spheres of heat shock group (Figure 4F). We analyzed stem cell markers after heat shock once every 2 days using Western blot assays, and found that OCT4, NANOG and SOX2 heavily decreased 2 days after heat shock (Figure 4G).

### 3.5. Hyperthermia Promotes GLI1 Degradation Through Ubiquitination–Proteasome Pathway

In order to clarify the mechanism of GLI1 degradation caused by heat shock, we used small molecule inhibitors to rescue GLI1 degradation. First, we tried MG132, a ubiquitination–proteasome pathway inhibitor, which rescued GLI1 degradation in two cell lines (Figure 5A), while chloroquine phosphate, the inhibitor of lysosome pathway can’t rescue GLI1 degradation induced by heat treatment (Figure 5B).

Figure 5Hyperthermia promotes GLI1 degradation through ubiquitination–proteasome pathway. (A, B) Hyperthermia‐induced degradation of GLI1 can be rescued by MG132 (A) but not CQ (B). KYSE70 and KYSE140 cells were treated with 10 μM MG132 for 4 h or 10 μM CQ for 24 h before heat treatment. Cells were collected immediately after heat treatment at 43°C for 30 min. (C, D) Hyperthermia‐induced degradation of GLI1 can be rescued by Apoptozole (AZ), an inhibitor of HSP70 (C) and Onalespib (Ona), an inhibitor of HSP90 (D). KYSE70 and KYSE140 cells were treated with 10 μM AZ or 10 μM Ona for 24 h before heat treatment. Cells were collected immediately after heat treatment at 43°C for 30 min. (E, F) Hyperthermia increases the ubiquitination levels of GLI1 and interaction with HSP70. KYSE70 cells were transfected with pCDNA3.1‐*myc-GLI1* plasmid, 48 h later collected immediately after heat treatment at 43°C for 30 min, and conducted immunoprecipitation using anti‐myc tag beads (E) or beads coupled HSP70 antibody (F), then detected myc tag; Ub and HSP70.

**Figure:**
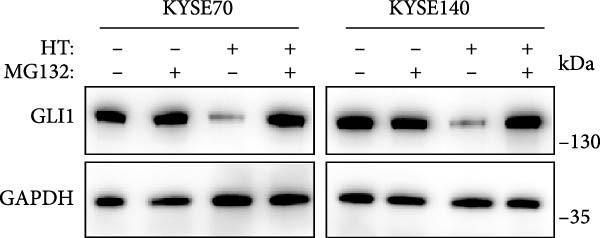
(A)

**Figure:**
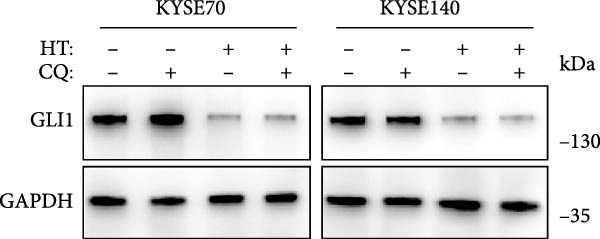
(B)

**Figure:**
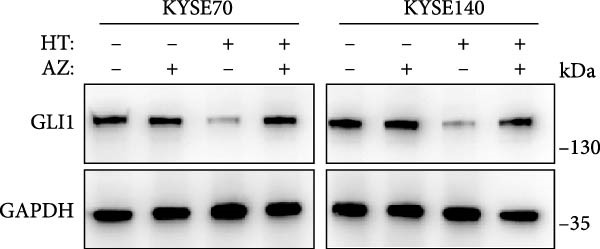
(C)

**Figure:**
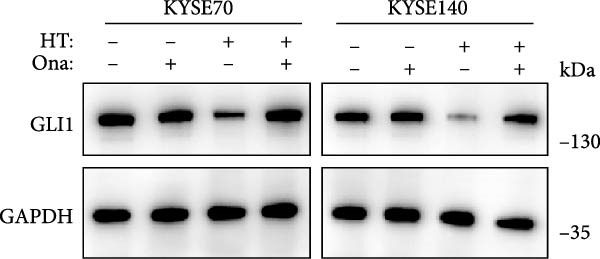
(D)

**Figure:**
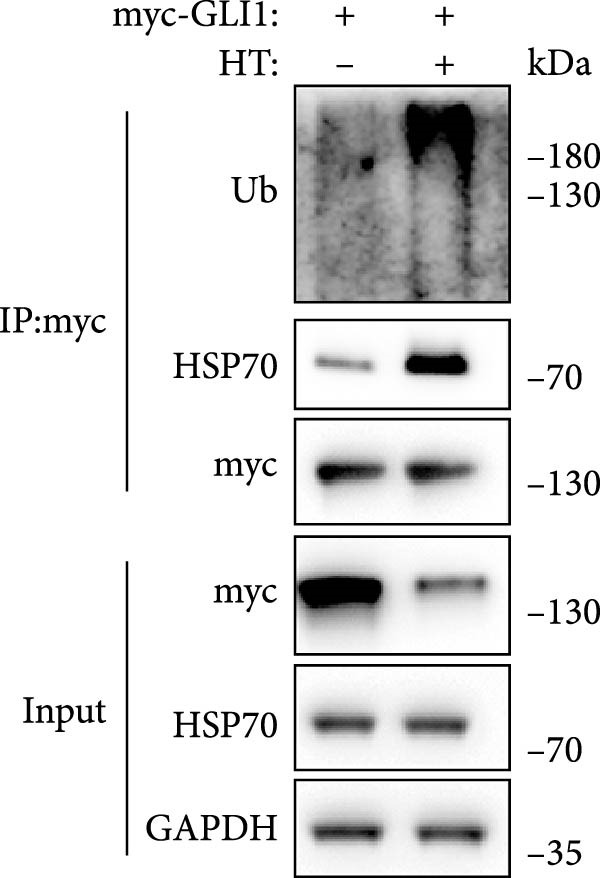
(E)

**Figure:**
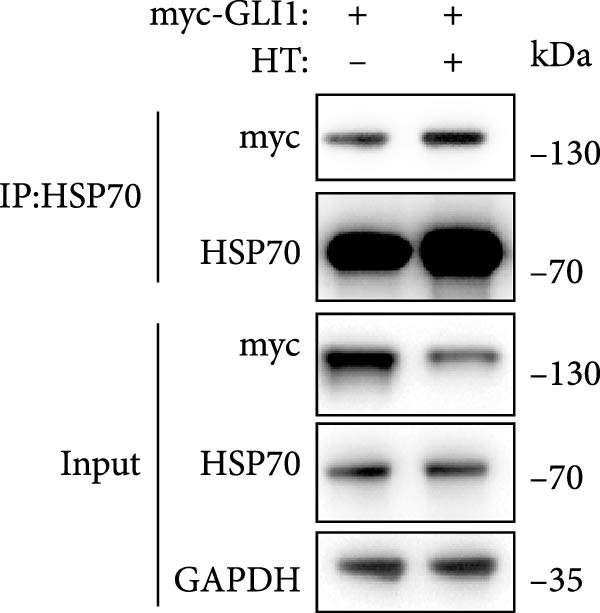
(F)

Since HSPs were reported can facilitate proteins ubiquitination level and lead to degradation of ubiquitinated proteins after heat shock. Second, we tried inhibitors of HSP70 (Apoptozole) and HSP90 (Onalespib) [28, 29]. Strikingly, both Apoptozole and Onalespib rescued GLI1 degradation caused by heat shock (Figure 5C,D).

As endogenous GLI1 level was relatively low, so it was hard to do immunoprecipitation experiment, then we express exogenous myc tagged GLI1 in KYSE70 cells and perform immunoprecipitation. Our data revealed heat shock increased GLI1 ubiquitination level and promote GLI1 interaction with HSP70 (Figure 5E,F).

## 4. Discussion

Canonical hedgehog signaling pathway is highly dependent on primary cilia, which function as a basics to transduce upstream of GLI1. Hedgehog pathway regulation mainly involves the control of *GLI1* expression through two paralogous transcription factors, GLI2/3. In the absence of hedgehog ligand, ciliary GPR161 promotes the partial proteolytic processing of GLI3 into transcription repressor (GLI3‐R). In the presence of hedgehog ligand, the ligand binds to its membrane receptor Patched, resulting in ciliary entry of SMO and the retrieval of ciliary Patched and GPR161 to enhance GLI activator production at the ciliary tip. GLI‐A then enters the nucleus to activate GLI1 expression. GLI1 expressed to high levels thereby activates its downstream genes. Canonical hedgehog has a vital role in embryo development, such as neural tube closure, limb formation [30]. While in many types of cancers, the activated hedgehog signaling is independent on primary cilia, as cancer cells lose primary cilia and upregulated GLI1 level directly, the detail mechanism is still poorly understood [31].

Hedgehog/GLI1 pathway is activated in part of ESCC, and may be correlated with ESCC progression, CSCs markers and EMT markers (Figure 1). Hedgehog/GLI1 pathway also correlated with therapy resistance and poor prognosis [32, 33].

Hyperthermia is an adjuvant therapy for many types of cancers with little side effects, which has been reported could target CSCs and suppress therapy resistant [34]. In this study, we found hyperthermia repress stemness of ESCC cells in vitro. Hyperthermia promotes GLI1 binds to HSPs, increase GLI1 ubiquitination level and degrade GLI1 through proteasome pathway. These data provide some evidences that how hyperthermia target CSCs. Our study also indicates that hyperthermia can be a promising therapy to GLI1 activated cancers.

We also consider the limitations of our study. In clinical practice, hyperthermia is always combined with surgery, radiotherapy and chemotherapy while our study investigated the effects of hyperthermia alone. Second, hyperthermia actions occur in the context of the tumor microenvironment where the immune system, fibroblast and extracellular matrix play an important role in the outcome of anticancer treatments. Our study involved athymic nude mice which have defective adaptive immunity, so this model was unable to properly assess the contribution of a fully intact immune system to hyperthermia responses.

## Conflicts of Interest

The authors declare no conflicts of interest.

## Author Contributions

Shenglei Li and Shichao Duan designed this project and wrote the paper. Hui Qin and Xiaole Li did the experiment and analyzed the data.

## Funding

This work was supported by the Henan Province Medical Science and Technology Research Plan Joint Project (Grant LHGJ20200352 for Hui Qin, China).

## Data Availability

The data are available upon request from the authors.
